# Targeting* O*-Acetyl-GD2 Ganglioside for Cancer Immunotherapy

**DOI:** 10.1155/2017/5604891

**Published:** 2017-01-05

**Authors:** Julien Fleurence, Sophie Fougeray, Meriem Bahri, Denis Cochonneau, Béatrice Clémenceau, François Paris, Andras Heczey, Stéphane Birklé

**Affiliations:** ^1^Inserm U892, Centre de Recherche en Cancérologie de Nantes-Angers, Institut de Recherche en Santé de l'Université de Nantes, Nantes, France; ^2^CNRS 6299, Centre de Recherche en Cancérologie de Nantes-Angers, Institut de Recherche en Santé de l'Université de Nantes, Nantes, France; ^3^Université de Nantes, UFR des Sciences Pharmaceutiques et Biologiques, Nantes, France; ^4^CHU Nantes, Hotel Dieu, Nantes, France; ^5^Texas Children's Cancer Center, Baylor College of Medicine, Houston, TX, USA

## Abstract

Target selection is a key feature in cancer immunotherapy, a promising field in cancer research. In this respect, gangliosides, a broad family of structurally related glycolipids, were suggested as potential targets for cancer immunotherapy based on their higher abundance in tumors when compared with the matched normal tissues. GD2 is the first ganglioside proven to be an effective target antigen for cancer immunotherapy with the regulatory approval of dinutuximab, a chimeric anti-GD2 therapeutic antibody. Although the therapeutic efficacy of anti-GD2 monoclonal antibodies is well documented, neuropathic pain may limit its application.* O*-Acetyl-GD2, the* O*-acetylated-derivative of GD2, has recently received attention as novel antigen to target GD2-positive cancers. The present paper examines the role of* O*-acetyl-GD2 in tumor biology as well as the available preclinical data of anti-*O*-acetyl-GD2 monoclonal antibodies. A discussion on the relevance of* O*-acetyl-GD2 in chimeric antigen receptor T cell therapy development is also included.

## 1. Introduction

Cancer immunotherapy comprises different strategies that use distinct effector mechanisms of the immune system to specifically target and eliminate tumor cells. Such strategies include specific monoclonal antibodies (mAbs), checkpoint inhibitors, cytokines, cancer vaccines, dendritic cell vaccines, tumor-infiltrating lymphocytes, and more recently genetically engineered T cells. Each one of these approaches holds promise, but their generalized success has been impaired by the paucity of specific tumor antigens resulting in suboptimal tumor efficacy and unpredictable side effects. Remarkably, the vast majority of these strategies have focused on protein antigens and recently mAbs recognizing cell surface gangliosides have recently proven to be effective for cancer therapeutic targets [1]. Gangliosides are sialic acid-enriched glycosphingolipids that contain at least one monosaccharide residue associated with a ceramide chain [2]. They are ubiquitously expressed in vertebrate tissues and are most abundant in the nervous system (for review see [3]). They exhibit a huge diversity due to the structural variations in both their oligosaccharide chain and ceramide moiety.

Almost 200 gangliosides species have been described, showing differences in the number, the order, and the linkage of the glycosyl and sialyl residues [4]. Sialic acid is a generic term for a member of a family of molecules presented by over 25 members. In addition, sialic acids can be further* O*-acetylated [5], de-*N*-acetylated [6], sulfated [7], or modified by lactonization [8]. The structural complexity and diversity further increase when variations of their ceramide anchor are taken into account. Such variations include the length, the saturation, and the hydroxylation of both the fatty acid chain and the long chain base. Remarkably, the diversity of gangliosides is generated by only a few glycosyltransferases and sialyltransferases that act within a combinatorial biosynthetic pathway [9]. After synthesis in the endoplasmic reticulum, the ceramide tail is transported to the Golgi apparatus. Then, glycosylation of ceramide occurs by membrane-bound glycosyltransferases and sialyltransferases that interact with their membrane-bound substrates [9]. Glycosylation and sialylation are coupled to exocytosis through the Golgi apparatus and transport vesicles to the plasma membrane [10]. After synthesis, gangliosides are present on the outer leaflet of the plasma membrane with their hydrophobic ceramide backbone anchored in the membrane and their hydrophilic carbohydrate residue projected into the extracellular environment. They are believed to be concentrated in ordered microdomains referred to as lipid rafts [11] where they can interact with different functional membrane proteins involved in cell adhesion and cell signaling [12]. Through these interactions, they can regulate crucial cell functions such as cell proliferation and apoptosis, adhesion, migration, and differentiation [13].

**Figure 1:**
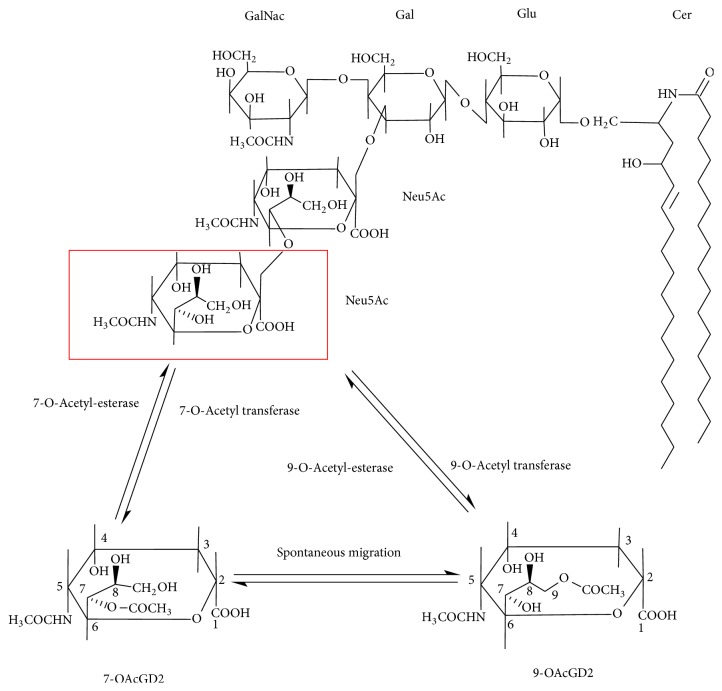
Structure of 9-*O*-acetyl-GD2.* O*-acetylated GD2 is constituted by a ceramide chain, which is anchored into plasma membrane, and a hydrophile chain. Oligosaccharide chain and* O*-acetylation are oriented to extracellular matrix.* O*-acetyl-GD2 is formed by the addition of an* O*-acetyl ester to the external sialic acid residue by a 9(7)-*O*-acetyl transferase.* O*-Acetyl esters located at the C7 position are mobile and can spontaneously migrate to the C9 position. They can be removed by 9(7)-acetyl esterases. Cer, ceramide; Gal, galactose; GalNAc,* N*-acetylgalactosamine; Neu5Ac,* N*-acetylneuraminic acid.

In addition, ganglioside concentration and distribution vary according to tissue, cell type, differentiation, and development stage [13]. It is therefore possible to define, for each cell type, a specific ganglioside profile, which is modified during embryogenesis, and ontogenesis [13]. Some ganglioside species further demonstrate very restricted expression in normal tissues and markedly enhanced expression in particular malignant tumor [14]. For example, ganglioside GD2 (Figure 1) is expressed at low concentration in the central nervous system [15], on peripheral nerves [15], skin melanocytes [15], and mesenchymal stem cells [16] in healthy adults. On the other hand, GD2 is overexpressed in tumors including neuroblastoma [15, 17], melanoma [18], small cell lung carcinoma [19], brain tumors [20], retinoblastoma [21], Ewing's sarcoma [22], and osteosarcoma [23]. Of note, the presence of GD2 was detected recently on breast cancer stem cells [24]. Cancer stem cells (CSC) represent a small population of tumor cells that are endowed with self-renewal and tumor-initiating capabilities [25]. Due to their inherent resilience, cancer stem cells are believed to underpin tumor recurrence and therapy resistance [25]. Thus, GD2 may also provide an effective target antigen for CSC immunotherapy. In fact, the National Cancer Institute pilot program for the prioritization of the most important cancer antigens ranks GD2 as number 12 out of 75 selected tumor antigens based on therapeutic function, immunogenicity, oncogenicity, specificity, expression level and percent of antigen-positive cells, stem cell expression, number of patients with antigen-positive cancers, number of epitopes, and cellular location of antigen expression [26] (Table 1). Of note, 3 other gangliosides (GD3, fucosyl-GM1, and GM3) were also selected, ranking between the positions 12 and 48 [26].

The clinical development of anti-GD2 mAbs for neuroblastoma patients originated from the discovery of two distinct murine anti-GD2 antibodies designated as 3F8 [27] and 14.18 [19], respectively. Dinutuximab, a.k.a. ch14.18, is a chimeric mouse/human IgG1 antibody obtained from the parental mouse IgG2a mAb 14G2a [28, 29] and named after the original mouse 14.18 IgG3 isotype [30]. In Europe, the cell line used for the production of ch14.18 antibody was changed. The plasmid encoding for ch14.18 antibody was recloned into CHO cells (ch14.18/CHO), and ch14.18/CHO antibody was designated as dinutuximab ß [31]. This antibody is currently under review by the EMA. Antibodies ch14.18 and 3F8 were further humanized to form hu14.18 and hu3F8, respectively [32, 33].

In 2010, Yu et al. reported a breakthrough randomized Phase 3 study in which the combination of cytokines IL-2 and GM-CSF with the anti-GD2 mAb ch14.18 (dinutuximab) showed a significant improvement in the event-free survival of high-risk neuroblastoma patients at the 2-year median follow-up [1]. These results led to the regulatory approval of dinutuximab by the FDA and the EMA in 2015 for the treatment of patients with high-risk neuroblastoma [34]. It is important to note that patients treated with anti-GD2 mAbs, such as dinutuximab, display dose-limiting acute toxicities, including hypotension, neuropathic pain, and fever upon antibody infusion [1]. These side effects are related to dinutuximab binding on GD2-positive sensitive nerve fibers followed by complement activation [32, 35]. Thus, improving the tolerance to anti-GD2 antibodies remains a key objective for GD2-targeted immunotherapies and different strategies are currently developed. A novel delivery method of ch14.18/CHO by continuous long-term infusion over 10 days is currently investigated in patients [36]. The binding to C1q can be abolished by incubation of the antibody at 56°C for 30 minutes [37] or by Fc-molecular engineering [32]. The most recent data challenge the use of IL-2 on the basis of a randomized trial using long-term infusion of dinutuximab ß combined with or without IL-2 [38]. In this trial, the addition of IL-2 increased pain and did not enhance anti-GD2 cytotoxicity, which therefore questioned the use of IL-2 with dinutuximab ß. Another strategy consists of targeting* O*-acetyl-GD2 that is not expressed on peripheral nerves [39]. In this review, we will present the* O*-acetyl-GD2 tumor antigen and its potential interest for antibody-based immunotherapy and chimeric antigen receptor cellular immunotherapy.

## 2. Structure and Physicochemical Properties of* O*-Acetyl-GD2 Ganglioside

*O*-Acetyl-GD2 is the* O*-acetyl derivative of GD2 ganglioside, in which the outer sialic acid residue is modified by an* O*-acetyl ester [40] (Figure 1).* O*-acetylated gangliosides arise by enzymatic transfer of* O*-acetyl group to the C7 and/or C9 hydroxyl groups on the glycerol-like side chain of a specific terminal *α*2-8 linked sialic acid residue. The commonest sialic acids found in ganglioside are* N*-acetyl-neuraminic acid and* N*-glycolyl-neuraminic acid [41].* N*-Glycolyl-neuraminic acid cannot be synthesized in man but can be expressed by human malignant tissue as a result of the metabolic incorporation of dietary* N*-glycolyl-neuraminic acid [42]. The addition of an* O*-acetyl group modifies several chemical properties of the ganglioside acceptor. For example,* O*-acetylation decreases the polarity and the hydrophobicity of the ganglioside but does not appear to affect its overall conformation [43]. The decrease in the polarity and the hydrophobicity is observable on thin-layer chromatography and high-performance liquid chromatography and can be used for separation of the* O*-acetylated ganglioside species. Of note, this group is very sensitive towards high temperature, alkaline pH, and naturally occurring esterases [44, 45]. The most striking chemical property of* O*-acetyl groups is the spontaneous migration from position 7 to position 9 observed in free* O*-acetylated sialic acids exposed to mild alkaline conditions [45]. Therefore, temperature and pH of the buffer should critically be maintained during sample collection and preparation, to avoid the loss and the migration of the* O*-acetyl group. This might explain the differences between authors describing the* O*-acetylation site in ganglioside. Thurin et al. [46] and Ostrander et al. [47] found that the* O*-acetyl group was located at the C9 position of the outer sialic acid of* O*-acetyl-GD3. Sjoberg et al. also found the* O*-acetyl group located at the C9 position of* O*-acetyl-GD2 [40]. In a later study, Ren et al. found that the* O*-acetyl ester was located at the C7 position of* O*-acetyl-GD3 [48]. However, some of these previous experiments have relied extensively on poorly resolved resonances in the ^1^H MNR spectra, which would be difficult in solving the resonance assignments for these compounds. In addition, in a follow-up paper, Manzi et al. found that the* O*-acetyl group located at both 7 and 9 positions [49]. This discrepancy might be related to the neuraminidase treatment and analysis of free sialic acid in their experimental procedure [49], which could facilitate the spontaneous migration of the* O*-acetyl group from the position C7 to C9 [45].

## 3. Metabolism of* O*-Acetyl-GD2 Ganglioside

The modification of ganglioside expression in oncogenesis is mainly associated with altered glycosyltransferase and sialyltransferase activities [50]. The level of* N*-acetylgalactosaminyltransferase I (GM2/GD2 synthase) activity is generally high in neuroectoderm-derived tumor cells, such as neuroblastomas and melanomas [50]. Although there is some evidence that posttranslational factors may influence enzyme activity [51] transcriptional regulation probably plays the major role [52]. Furukawa et al. evidenced that the GM2/GD2 synthase gene has three transcription initiation sites and further revealed that the regulatory mechanisms for each transcription of this gene were more complex than expected [52]. Not much information is now available on this point. The high expression of GM2/GD2 synthase leads to the accumulation of GD2 ganglioside in neuroblastoma cells [50]. Accumulation of tumor-associated gangliosides is further associated with aberrant sialylation of their sialic acid content. As mentioned above, human tumor cells can incorporate dietary* N*-glycolyl-neuraminic acid in their gangliosides [42], but also can* O*-acetylate their outmost sialic acid residue [5].

Current models suggest that two types of enzymes are involved in the metabolism of* O*-acetylated sialic acids:* O*-acetyl transferase [53] and* O*-acetyl esterase [54]. As mentioned above, the initial site of the* O*-acetylation remains debated [45]. Apparently, the* O*-acetyl transferase activity transfers the* O*-acetyl group to carbon C7 [55]. Once located at cell surface and exposed to higher pH, 7-*O*-acetyl group may migrate to position 9, explaining part of the above discrepancies observed between authors. Nevertheless, the* O*-acetyl transferase reaction appears to occur concertedly with sialyltransferases in the Golgi apparatus, which synthesizes the ganglioside acceptor, and to be acetyl-coenzyme A dependent [56]. Singularly, the relevant* O*-acetyl transferase responsible for ganglioside* O*-acetylation has not yet been isolated or identified. No gangliosidosis due to a defect of the* O*-acetyl transferase has been found so far. The direct purification of the* O*-acetyl transferase activity seems to be affected by an inherent sensitivity to membrane solubilization and remains difficult [56]. Different attempts for cloning the cDNA of the* O*-acetyl transferase by heterologous cell-cDNA library expression cloning resulted in the identification of proteins inducing* O*-acetylation but that are not* O*-acetyl transferases specific for ganglioside [57–61]. Of note, Vandamme-Feldhaus and Schauer found the* O*-acetyl transferase activity in fraction associated with membranes [62]. Thus, in the most recent attempt to isolate a true* O*-acetyl transferase Arming et al. screened the human genome database targeting the gene candidates that would fit the proposed model of a membrane-bound* O*-acetyltransferase located in the Golgi apparatus [63]. Their search resolved the candidate gene CASD1 (capsule structure 1 domain containing 1) [63]. Baumann et al., in the follow-up paper, gathered stronger evidence of the involvement of CAS 1 in sialic acid* O*-acetylation using CRISPR/Cas 9 gene edition and recombinant CAS 1 protein [64]. The authors were further able to* O*-acetylate free sialic acid molecule using a soluble recombinant CAS 1 protein [64]. However, the authors did not provide evidence that the recombinant form of CAS1 protein they used in their study was also able to transfer the* O*-acetyl group directly to the terminal sialic acid of GD3 ganglioside. An alternative explanation is that* O*-acetylation of sialic acid actually takes place at the sugar nucleotide level. CMP-*O*-acetyl-sialate could then act as a donor for synthesis of* O*-acetyl GD3 from GM3, by the action of GD3 synthase.

**Figure 2:**
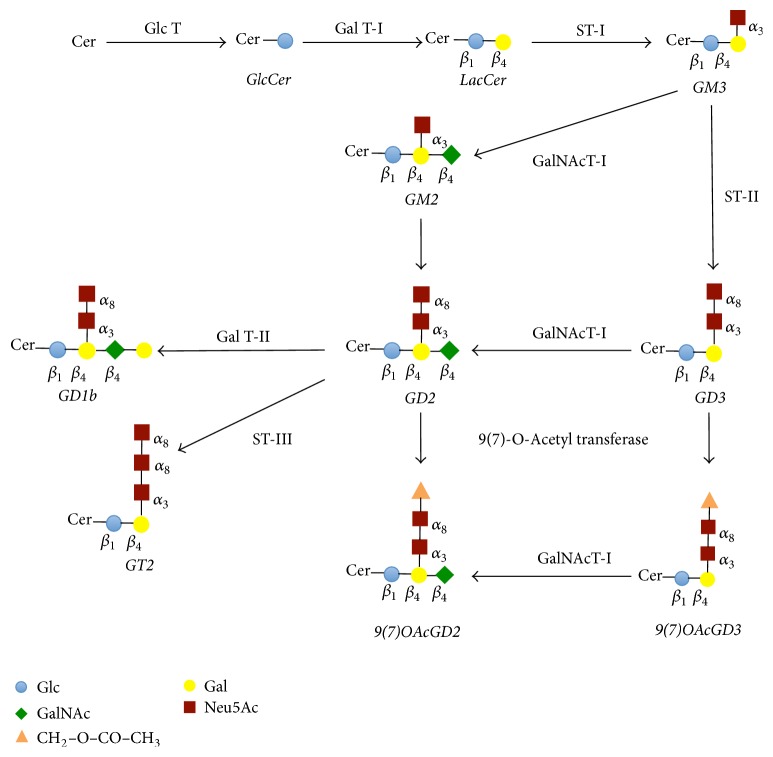
Schematic representation of the main pathway of* O*-acetyl-GD2 ganglioside biosynthesis. GD2 ganglioside is synthesized by the action of* N*-acetyl-galactosyltransferase I, which transfers* N*-acetyl-galactosaminyl residue from UDP-*N*-acetyl-galactosamine to GD3 [66]. GD2 can be also formed by the action of alpha 2–8 sialyltransferase II, which transfers a sialic acid residue from CMP-sialic acid to GM2 [66]. After synthesis, GD2 can be converted into either GD1b or GT2. GD1b is formed by the action of galactosyltransferase II that transfers a galactose residue from UDP-galactose to GD2. GT2 is synthesized by the action of sialyltransferase III that transfers a sialic acid residue from CMP-sialic acid to GD2. Then, the* O*-acetyl group addition occurs in a postsynthetic fashion [66]. Thus,* O*-acetyl-GD2 can be synthesized either by the action of sialate-*O*-acetyltransferase, which transfers the* O*-acetyl group to GD2, or by the action of* N*-acetyl-galactosyltransferase I, which transfers* N*-acetyl-galactosaminyl residue from UDP-*N*-acetyl-galactosamine to* O*-acetyl-GD3. Cer, ceramide; GlcCer, glucosylceramide; LacCer, lactosylceramide; Gal, galactose; Glc, glucose; GalNAc,* N*-acetylgalactosamine; Neu5Ac,* N*-acetylneuraminic acid; Glc T, glucosyltransferase; Gal T, galactosyltransferase; ST, sialyltransferase; GalNacT,* N*-acetyl-galactosaminyltransferase; CASD 1, Cas 1 domain containing 9(7)-*O*-acetyl transferase.

Surprisingly,* O*-acetyl-GD2 is concomitantly expressed with GD2 at the tumor cell surface. The ratio between the amount of* O*-acetyl-GD2 and the amount of GD2 varies from 10% up to 50% [39, 65]. This observation suggests another point of control in* O*-acetyl-GD2 biosynthesis. In fact, any given step in ganglioside biosynthesis requires the colocalization of the appropriate acceptor, sugar nucleotide transporter, and glycosyl-transferase activity. Thus, the synthesis of* O*-acetyl-GD2 can be regulated by the amount of acetyl-CoA concentrations within the Golgi apparatus [56]. The use of biochemical compounds that selectively block the transport from the endoplasmic reticulum to the Golgi apparatus further suggested that* O*-acetyl-GD2 can be synthesized from either* O*-acetyl-GD3 or GD2, in respect to either the nature or the localization of the glycosyltransferases expressed by the cells [40, 66]. In addition, the above mechanisms do not further exclude a possible turnover of* O*-acetyl esters bound to sialic acids of gangliosides controlled by sialate-*O*-acetylesterases [67]. Hence, the expression of* O*-acetyl-GD2 in a cell type may be the result of the conjunction of, at least, four parameters (Figure 2): the balance between two enzymatic systems,* O*-acetyl transferase and* O*-acetyl esterase; the activity of the* N*-acetyl-galactosaminyltransferase I that synthesizes* O*-acetyl-GD2 from* O*-acetyl-GD3; the activity of the *α*2-8 sialyltransferase II that forms GD2 from GM2; and the activity of the galactosyl-transferase II that forms GD1b from GD2 (Figure 2). Given the complexity of this biosynthetic model, the clarification of the mechanisms that regulate the expression of* O*-acetyl-GD2 remains challenging. This complexity further delays the elucidation of* O*-acetyl-GD2 functional role in tumorigenesis through gene edition approaches and its potential interest as a prognostic marker. Given that, mAbs specific for* O*-acetyl-GD2 remain the best reagents available to study* O*-acetyl-GD2 functions in tumor progression.

## 4. Functional Aspects of* O*-Acetyl-GD2

In the absence of the characterization of a specific ganglioside* O*-acetyl transferase, the elucidation of the functional implications of* O*-acetylated gangliosides remains, somehow, a conundrum. The most elaborately studied* O*-acetylated ganglioside remains* O*-acetyl-GD3 because it was the first identified member of this family [5]. In the central nervous system,* O*-acetyl-GD3 appears to be involved in the extension of neuronal growth cone and neurite extension since these phenomena can be inhibited in vitro by anti-*O*-acetyl-GD3 mAbs [68]. Elimination of* O*-acetyl-GD3 expression in the retina and adrenal of transgenic mice gave variable abnormalities in development [69]. Postnatally,* O-*acetyl-GD3 species defines the epitope for anti-CDw60 antibodies in human lymphocytes [70], although similar structure on glycoprotein glycans also contributes [71]. Additional evidence indicates that* O*-acetyl-GD3 is involved as cell surface structures in lymphocyte activation [72, 73] and as intracellular substances in the regulation of apoptosis [74, 75]. In tumor cells, such as melanoma,* O*-acetyl-GD3 seems to contribute to tumor cell proliferation [76]. In glioblastoma, it may also promote tumor cell survival [77]. Similar observations were reported in acute lymphoblastic leukemia cells [74, 78] in which* O-*acetyl-GD3 seems further to contribute to their drug resistance capacity [79]. Importantly, the evidence for the antiapoptotic functions of* O*-acetyl-GD3 in the above studies remains indirect. In addition to the effects of mAbs specific for* O*-acetyl-GD3 [68, 72, 73, 80], they consist of the effects induced by the addition of* O*-acetyl-GD3 into cells [74, 75] and the effect of the viral 9-*O*-acetylesterase [69, 76, 77, 79].

In contrast to* O*-acetyl-GD3, very little is known about the biological role of* O*-acetyl-GD2. We showed that* O-*acetyl-GD2 is a proapoptotic constituent in tumor cells activated on binding with hostile antibodies [80]. While* O*-acetyl-GD2 can transmit signals resulting in apoptosis, the precise mechanisms induced by the binding of* O*-acetyl-GD2 antibody to* O*-acetyl-GD2-expressing tumor cells leading to apoptosis require further investigation. In the case of GD2, initial indications suggest that anti-GD2 mAbs induce apoptosis of SCLC cells by interfering with the association of GD2 ganglioside to ß1-integrin and focal adhesion kinase, which triggers the p38-dependent apoptotic pathway [81]. Data obtained with antibody 14G2a in neuroblastoma cells further suggested that apoptosis induced by anti-GD2 mAbs resulted in activation of both extrinsic and intrinsic caspase-dependent and caspase-independent apoptotic pathways [82]. In melanoma cells binding of antibody 3F8 resulted in the activation of caspases 3, 7, and 8, the release of cytochrome c and AIF, and the downregulation of both survivin and Apaf-1 without the activation of caspase 9 [83]. Of note, both mAbs 14G2a and 3F8 cross-react with* O*-acetyl-GD2 [40, 65]. Therefore, it is not excluded that the mechanisms evidenced in these studies also involved* O-*acetyl-GD2. Hence,* O*-acetyl-GD2 may behave very similarly to GD2 in mediating apoptosis in the GD2/*O*AcGD2-expressing tumor cells [81, 83–85], in disagreement with the antiapoptotic role evidenced for* O-*acetyl-GD3 [74, 77–79].

## 5. Distribution of Ganglioside* O*-Acetyl-GD2 in Normal Tissues

Beside many unknowns regarding its biosynthesis and biological roles,* O*-acetyl-GD2 provides an opportunity to develop new immunotherapeutic strategies based on therapeutic antibodies, cancer vaccines, and adoptive transfer of lymphocytes, genetically engineered to acquire tumor cell specificity. Immunotherapies of cancers exploit the fact that tumor cells often expressed antigen molecules on their surface that can be detected by specific antibodies. Such molecules are known as tumor-associated antigens (TAA). When the same or related antigenic determinant is expressed on human cells or tissues other than the intended target tissue, binding of the antibody to this tissue may be observed. Nontarget tissue binding may have serious consequences, leading to on-target off-tumor toxicities. Accordingly, anti-TAA mAbs may cross-react with other antigens expressed by normal tissues. It is therefore advisable to establish the TAA and the cross-reactivity profile of the anti-TAA antibody before initiating any experiment. The most common approach to assess antibody cross-reactivity is immunohistochemistry or immunofluorescence using animal and human tissues to allow comparison of the results. Within the U.S. Food and Drug Administration (FDA), the Center for Biologics Evaluation and Research (CBER) provides, and regularly updates, “points to consider (PTC) in the manufacture and testing of mAb products for human use” (http://www.fda.gov/cber/gdlns/ptc mab.pdf) [86].

Thus, our group studied the* O*-acetyl-GD2 distribution in healthy tissues by immunohistochemistry using the anti-*O*-acetyl-GD2 mAb 8B6 [39] according to the FDA guidelines [86]. We found that, in contrast to GD2,* O*-acetyl-GD2 was not detected on peripheral nerves [39]. As mentioned earlier, the therapeutic use of anti-GD2 mAbs is associated with important neurotoxic effects in patients, due to the cross-reactivity of anti-GD2 mAbs normal nerve fibers [32, 35]. Hence, our results suggest that mAbs specific for* O*-acetyl-GD2 should be less toxic because they do not bind to peripheral nerves [39]. Some other side effects observed in patients after anti-GD2 mAb infusions include hematopoietic suppression [16] and a syndrome of inappropriate antidiuretic hormone [35]. These side effects may be related to the possible immune recognition of GD2 on mesenchymal stromal cells in the marrow microenvironment and the anti-GD2 mAb cross-reactivity with the posterior lobe of the pituitary gland [35]. There was also slight reactivity with Purkinje cells, the Bergmann glia in the cerebellum, and the dorsal horns in the spinal cord. Furthermore, mAb 8B6 did not show any binding either to mesenchymal stromal cells in the bone marrow or to the posterior lobe of the pituitary gland. These data indicate that mAb 8B6 presents a very interesting safety reactivity profile for its clinical use.

## 6. Distribution of Ganglioside* O*-Acetyl-GD2 in Malignant Tissues

We examined the immunohistochemical* O*-acetyl-GD2 expression in a number of malignant tissues and found that mAb 8B6 showed strong reactivity with neuroectodermic tumor biopsy tissues, such as melanoma and neuroblastoma [39] similar to previous investigations [40, 65]. In vitro data further demonstrated a high expression of* O*-acetyl-GD2 at the tumor cell surface by Scatchard analysis, with an average of sites/cell ranging from 50,000 sites/cell up to 5 × 10^6^ sites/cell [39]. Importantly, we showed that the amount of* O-*acetyl-GD2 molecules present at the cell surface was comparable, though lower, to that of GD2 epitope [39]. Taken together, these data suggest that GD2 is differentially acetylated in normal and tumor tissue and that normal tissues expressing GD2 may not express* O-*acetyl-GD2. This prompted us to investigate the expression of* O*-acetyl-GD2 in glioblastoma multiforme (GBM) [87], a cancer that is known to express GD2 [20]. We confirmed the presence of* O*-acetyl-GD2 on GBM biopsies obtained after surgical resection. We also demonstrated high* O*-acetyl-GD2 expression on GBM cell lines and patient-derived tumor cells [87]. Of note, GBM is a lethal and therapy-resistant brain cancer comprised of several tumor cell subpopulations, including glioblastoma stem cells (GSCs) [88]. As mentioned above, these cells demonstrate resistance to current chemoradiotherapeutic options and are believed to reinitiate malignancies after initial responses to therapies [25, 89]. Therefore, new therapeutic approaches must consider eliminating both GSCs and the entire bulk of the tumor. The markers used to define GSCs have been however in constant evolution since the evidence of a CD133-positive subpopulation in glioblastoma that retained stem-like properties and were capable of establishing glioblastoma tumors in mice with similar phenotype to those of the patients [88]. Interestingly, Battula et al. [24] reported that in breast carcinomas ganglioside GD2 is a marker of breast carcinoma stem cells capable of initiating tumors at a higher frequency than GD2-negative cells. Of note, they used in their study the anti-GD2 mouse mAb 14G2a that, as mentioned earlier, cross-reacts with* O*-acetyl-GD2 ganglioside [40]. Thus,* O*-acetyl-GD2 may be also expressed by GD2-positive cancer stem cells, if not involved in cancer cell stemness. However, over the past decade, multiple other cancer stem cell markers have been identified challenging a reliable identification of tumor-specific antigen to be used for anti-GSC immunotherapies [90]. This question is currently studied in our laboratory.

## 7. Immunogenicity of* O*-Acetyl-GD2

Being a carbohydrate antigen,* O*-acetyl-GD2 possesses identical biochemical structures between species, even in very distant species. Thus, specific antibody specific for* O*-acetyl-GD2 expressed by human tumor cells can easily cross-react with the antigen in animals. Moreover, it is a T cell-independent antigen, an interesting characteristic in tumors that are either poorly immunogenic for T cells or that evade T cells mainly by downregulating or losing human leukocyte antigen (HLA) expression. However, one of the most challenges to generate mAb against such antigen remains the low efficiency of the ganglioside immunization [91, 92]. This is due to poor immunogenicity of carbohydrate antigens. Repeated injection of gangliosides alone in human or in mouse is not enough for inducing the synthesis of anti-ganglioside antibodies in vaccinated host. The reason for their poor immunogenicity may be due, in part, to the phylogenetic conservation of ganglioside structures resulting in tolerance. In addition, carbohydrate antigens generally invoke a T cell-independent immune response, during which IgM can be typically driven into IgG3 in naive mice. For this reason, gangliosides are generally classified as T-independent type 2 antigen. Activation of specific B cells to these antigens in the absence of MHC class II-restricted T cell help requires antigen receptor cross-linking. However, this cannot be achieved with small antigens such as gangliosides. Thus, optimization of the immunization protocols against* O*-acetylated gangliosides is required to generate high-titers of both affinity-matured and class-switched antibodies for the production of mAbs as tool for research, diagnosis, and therapy. Another approach to circumvent the limitation imposed by the intrinsic immunogenicity of ganglioside consists in the in vitro isolation of anti-ganglioside antibody fragments based on the screening of a large library followed by refined mutagenesis [93, 94].

Given all these limitations, our group used whole-cell immunization protocol to generate the mouse mAb 8B6 specific for* O*-acetylated-GD2 [95]. The antibody 8B6 (IgG3, kappa) was derived from A/J mice immunized with LAN-1 neuroblastoma cells [95]. The specificity of antibody 8B6 for* O*-acetyl-GD2 was confirmed by immunostaining on thin-layer chromatography. When tested on total neuroblastoma ganglioside, antibody 8B6 stained exclusively* O*-acetyl-GD2 [95]. We also calculated the affinity of antibody 8B6 for* O*-acetylated GD2 to be 32 nM [39]. In addition, we evidenced the structure of the variable VH and VL gene encoding 8B6 hybridoma [95]. Surprisingly, the VH segment of hybridoma 8B6 revealed the presence of somatic mutations, suggesting the occurrence of an affinity maturation process [95].

As mentioned above, targeting GD2 has been a matter of concern due to the possibility of inducing autoimmune responses against peripheral nerves. Indeed, in most neuropathies of immunological origin, endogenous gangliosides have been shown to be the target of the autoimmune reactions [96]. Thus, the tumor-specific expression of* O*-acetyl-GD2 in some human tumors suggests that the induction of an effective immune response against these antigens may be useful for patients with antigen-positive tumors. The absence of expression in normal tissue allows for increased immune responses to immunization while precluding self-targeted reactions.

An original strategy to elicit an immune response against ganglioside antigen relies on the development of anti-idiotypic mAbs as antigen surrogates. Such an antibody represents the internal image of the antigen. Thus, anti-idiotype antibodies can act as antigens, inducing a response against the original antigen. A remarkable advantage of anti-idiotype mAbs is the fact that the constant regions of the anti-idiotypic antibody can serve to boost antitumor immune responses [97]. This is a useful strategy to induce an antibody response towards a ganglioside, which is a weak immunogenic molecule in itself. Furthermore, anti-idiotypic antibody provides important tools for the immune monitoring of clinical trial with anti-gangliosides antibodies. An example of a vaccine development using this strategy is given by racotumomab, a murine anti-idiotypic antibody raised from the anti-de-*N*-glycolyl-GM3 mAb P3 [98]. Racotumomab had been used in several clinical trials and its safety and efficacy were assessed in different tumor localizations: melanoma, breast, and lung cancers. More recently, there was a specific interest in pediatric tumors expressing* N*-glycosylated gangliosides. Racotumomab has now reached the Phase III clinical trials with possible indication in lung cancer [99] and a possible extent to pediatric tumors [100]. Anti-idiotypic mAbs are conceptually easy to generate after immunization of mouse with the parental mAb. However, generating one remains challenging since it requires tight selection, as most of the epitopes on the parental mAb will be irrelevant.

## 8. Passive Immunotherapy with Anti-*O*-acetyl-GD2 mAbs

MAbs have demonstrated their potential as anticancer therapies. Since the first approval of rituximab—a chimeric mAb targeting CD20—for the treatment of patients with lymphoma in 1994, more than 10 therapeutic antibodies have been approved for passive immunotherapy of cancer; all of them are directed against protein antigens. Although a long list of tumor-associated carbohydrate antigens has been identified over the past two decades, many of the clinical trials did not proceed beyond Phase I/II studies. In this regard, with the approval of dinutuximab in 2015, GD2 became the first glycan antigen proven to be effective target antigen for cancer immunotherapy [1, 34]. Three other immunotherapeutic strategies are currently developed to enhance the potency of anti-GD2 immunotherapy such as immunocytokines, bispecific antibodies, GD2-specific chimeric antigen receptor T cells, and GD2 vaccines. However, given the tissue distribution pattern of* O*-acetyl-GD2, the* O*-acetyl derivative of GD2 provides a potential opportunity to develop safer immunotherapeutic strategies.

Our group analyzed the antitumor activity and the preclinical toxicity of the mAb 8B6 using different animal models, according to the putative mechanisms of action of dinutuximab. Dinutuximab (ch14.18) and other anti-GD2 antibodies have been shown to induce ADCC as well as CDC in GD2-expressing cell lines [31, 101]. However, ADCC is considered as an important clinical mechanism for anti-GD2 immunotherapy [102, 103]. In this respect, anti-*O*-acetyl-GD2 mAbs seem to be particularly effective as compared to anti-GD2 antibodies in mediating ADCC against* O*-acetyl-GD2-expressing tumor cells [39, 87, 104]. We also found that anti-*O*-acetyl-GD2 mAb antibodies induce significant CDC in addition to ADCC in vitro [39]. Since anti-GD2 antibody CDC activity is believed to be responsible for the pain side effects [32] an overdrive of CDC may also be desirable to further enhance anti-*O*-acetyl-GD2 mAbs antitumor activity.

As mentioned earlier, our group reported a possible role of* O*-acetyl-GD2 in tumor cell death with the mAb 8B6, in addition to its immunological cytotoxicity [80]. The cell death induced by the binding of antibody 8B6 on the target cancer cells triggered the p38-signaling pathway. This was correlated with a cycle arrest of the cell, an increase in p21 protein levels, and the expression of apoptosis-associated proteins such as phospho-p38, BAX, cytochrome c in cytoplasm, and cleaved caspase 3 [80]. Peculiarly, this mechanism of killing requires a far higher concentration of antibody than is typically required for ADCC and CDC but not one that is compatible with serum concentration commonly observed in patients with melanoma and neuroblastoma following the infusion of anti-GD2 mAbs [105]. The precise mechanisms by which antibodies specific for* O*-acetyl-GD2 trigger tumor cell death still require clarification. While they seem to be influenced by* O*-acetyl-GD2 density at the tumor cell surface, they are also influenced by the antibody isotype [104] similarly to anti-GD2 mAbs [83]. Thus, the proapoptotic activity of anti-*O*-acetyl-GD2 mAbs cannot be fully explained by their sole antigen recognition activity. Finally, we demonstrated the implication of this proapoptotic activity on the antitumor efficacy of antibody 8B6 in vivo in human neuroblastoma IMR5 tumor-bearing NOD-SCID mice, which lack NK cell-cytotoxic activity and circulating complement [80]. From a clinical stand point, the apoptosis inducing activity of anti-*O*-acetyl-GD2 mAb 8B6 seems, however, very promising when applied to cancer therapy. This property may be important in the treatment of tumors that have evolved complex mechanisms to protect themselves from ADCC and CDC.

To facilitate clinical development of therapeutic antibodies targeting* O*-acetyl-GD2, our group also developed a mouse/human IgG1 chimeric version of antibody 8B6 [104]. The chimeric IgG1 c.8B6 antibody was obtained from the mouse mAb 8B6 to* O*-acetyl-GD2 and expressed in CHO-S cells. It retains the same antigen binding affinity and specificity as its parental mouse mAb [104]. We further showed that chimeric 8B6 antibody was able to inhibit NXS2 liver metastasis as efficiently as dinutuximab (ch14.18) [104]. More importantly, we performed studies in rat demonstrating that intravenous c.8B6 treatment did not induce allodynia as compared to ch14.18 [104]. In addition to the absence of* O*-acetyl-GD2 expression on nerve fibers, the lack of allodynic properties of anti-GD2 antibodies provided an important rationale for the clinical application of chimeric 8B6 antibody in patients with* O*-acetyl-GD2-expressing tumors. Clinical trial of mAb c.8B6 is eagerly awaited.

## 9. Adoptive Immunotherapy with* O*-Acetyl-GD2-Specific CARs

**Figure 3:**
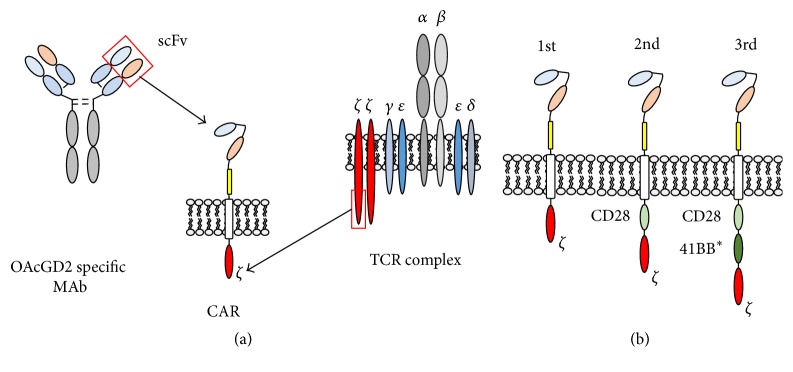
Structure of* O*-acetyl-GD2 CAR examples. (a) An* O-*acetyl-GD2-specific mAb derived scFv is linked through a spacer and a transmembrane domain to the T cell receptor (TCR) complex CD3*ζ* chain intracellular signaling domain. (b) Additional costimulatory endodomains are shown in 2nd- and 3rd-generation CARs derived from CD28 or CD28 and 41BB, respectively. ^*∗*^41BB may be exchanged to other domains such as OX40 or ICOS.

Chimeric antigen receptor (CAR) based adoptive immunotherapy is an attractive approach to treat patients with cancer as this strategy can combine the specificity of mAb with the active biodistribution, expansion potential, long-term persistence, and cytotoxic function of effector immune-cells [106, 107]. The technology holds great promise for cancer therapy and has generated breakthrough responses in recent years including 91% complete remission rates in patients with CD19-positive B cell acute lymphoblastic leukemia and complete tumor regression in patients with bulky CD19-positive B cell lymphoma [108–111]. CAR expressing effector cells can recognize tumor cells in a Major Histocompatibility Complex/Human Leukocyte Antigen (MHC-HLA) independent manner; thus the recognition of tumor cells is not affected by two key tumor escape mechanisms: downregulation of MHC Class 1 or Class 2 molecules and altered antigen processing [106, 112, 113]. CARs are derived from a single chain fragment variable (scFv) of mAb linked through a spacer and transmembrane domains to intracellular signaling domains forming a unique fusion protein (Figure 3(a)) [114]. While the scFv binds the tumor antigen, the signaling domains are responsible for initiating the activating signals in effector cells leading to tumor elimination and expansion of CAR expressing cells resulting in long-term tumor surveillance. Given the promising potential of CAR based therapies, the development of* O*-acetyl-GD2-specific CARs may provide an effective therapeutic approach for patients with several solid malignancies.

Results from early phase clinical studies showed that the incorporation of endodomains from costimulatory receptors (Figure 3(b)) is critical for potent antitumor effect [106, 115–117]. The first GD2-CAR was developed by Rossig et al. based on scFv derived from the GD2-specific mAb 14G2a [118, 119] and was tested in Phase 1 clinical study [120, 121]. This trial evaluated a 1st-generation GD2-specific CAR in children with relapsing/refractory neuroblastoma and compared the safety, persistence, and antitumor efficacy of two effector cell populations: activated T cells (ATC) and Epstein-Barr virus specific cytotoxic lymphocytes (EBV-CTLs) [120]. No dose-limiting toxicity was found, the therapy was well tolerated, and some patients achieved long-term complete tumor regression. Interestingly, the GD2-CAR expressing EBV-CTLs persisted significantly longer and not surprisingly long-term effector cell persistence was also associated with better antitumor efficacy [121]. Given the limited persistence of adoptively transferred GD2-CAR T cells, a 3rd-generation GD2-CAR incorporating CD28 and OX40 costimulatory endodomains was developed by Pulè et al. [122]. A Phase 1 clinical trial recently completed accrual testing this 3rd-generation CAR in children with relapsed/refractory neuroblastoma and the results of this study are expected in 2016 (NCT01822652). Another Phase 1 study (NCT01953900) is open for accrual using the same 3rd-generation CAR construct expressed in varicella zoster virus specific CTLs (VZV-CTLs) testing the approach in patients with osteosarcoma. This strategy will assess the effect of VZV vaccination on the in vivo expansion and persistence of transgenic VZV-CTLs.

Given the restricted expression of* O*-acetyl-GD2 on cancer cell and its presence on certain cancer stem cells, developing CARs to target* O*-acetyl-GD2 is a promising approach to help patients. To find the CAR constructs with the antitumor potential resulting in complete regression of* O*-acetyl-GD2-expressing solid tumors, the construct and the effector cell type will have to be carefully selected for the specific tumor type and several variables must be carefully evaluated.

The scFvs, particularly those targeting GD2, can induce clustering of CARs resulting in chronic tonic signaling which can exhaust effector cells leading to decreased cytotoxic capacity, proliferation, and persistence after adoptive transfer [123]. Therefore,* O*-acetyl-GD2-CAR should derive from scFv with minimal tonic signaling induction. Several clinical trials have detected B and T cell responses against mouse-derived scFvs, ultimately leading to the elimination of CAR T cells and therefore limiting their ability to effectively eliminate tumors and provide long-term tumor surveillance. Thus, the use of full mouse-scFv can negatively affect the efficacy of adoptive cell therapies by inducing immune responses [124–126]. Fully human or at least humanized scFvs are likely better candidates for CAR development as these scFvs are the least likely to induce an adoptive immune response.

In addition, to find the optimal scFv, the spacer and transmembrane domains need attention as well. Accumulating evidence suggests that the spacer region of the CAR is not a simple bridge to the transmembrane domain; rather it plays an important role in building and maintaining the immunologic synapse and therefore providing the optimal activating signals for the effector cells. Studies have shown that the optimal length of the spacer region is target antigen-dependent and different tumor targets require different space lengths to optimize the CAR function [127, 128]. The spacer length may need to be adjusted specific antigens to resemble the distance between effector and target cells similarly to the physiologic immune synapse. A recent publication confirmed the importance of CAR design by showing that, for GD2, the IgG Fc region appeared to be the optimal spacer [129]. However, with this type of spacer there is a theoretical risk of engagement of CAR expressing T cells with FcR-expressing cells resulting in both off-target toxicity of myeloid cells and diversion of the CAR T cells from their intended effector function [130, 131].

The transmembrane domain is not only responsible for keeping the CAR membrane bound, but also important for stable CAR expression. Transmembrane domains can be derived from several transmembrane proteins including CD3*ζ*, CD4, CD8, or CD28 molecules [113]. The initial CAR design incorporated CD3*ζ* derived transmembrane sequences. On one hand this domain allows incorporation of the CAR into the T cell receptor complex and improves signaling [132, 133]. However, CAR cell surface expression is less stable compared to CD28 transmembrane domain [117]. It is not clear which transmembrane domain is optimal for CAR based therapies and testing distinct versions of this domain in the context of a specific target antigen and various effector cell population may be necessary. In addition, the incorporation of costimulatory endodomains into the CAR structure can significantly improve the antitumor potential of effector cells [106]. These endodomains can derive, for example, from CD28, OX40, ICOS, or 4-1BB [110, 115, 117, 122, 134–137] and the type and combination of these endodomains may require further testing depending on the effector cell used to express the* O*-acetyl-GD2-specific CAR.

The role and difference of specific effector cell population have gained more attention in recent years as another important aspect for effective adoptive immunotherapy. In the context of 1st-generation CARs, virus specific T cells (VSTs) can provide additional costimulatory signals and improve CAR T cell persistence and antitumor functions [120, 121, 138]. Manufacturing of CAR-VSTs is significantly more complex compared to polyclonal activated T cells (i.e., T cells stimulated with CD3-CD28 beads and IL-2) and it is still to be elucidated whether VSTs provide any advantage over activated T cells when 2nd- or 3rd-generation CARs are used.

To date, polyclonal activated T cells have been the most common CAR expressing effectors, but other cell populations may become promising platforms for CAR based immunotherapy. Effector T cells (CD45RO-pos, CD62L-neg) are a logical choice for immunotherapy due to their high cytotoxic potential; however, these cells have limited persistence which is essential for adoptive immunotherapy. Long-lived, central memory or stem cell memory T cells have significantly better expansion potential, persist longer, and have been shown to induce superior antitumor effects in preclinical studies making this population particularly attractive for adoptive immunotherapy [121, 139–141]. Other cell populations such as natural killer T cells (NKTs) hold a great promise for the treatment of neuroblastoma. NKTs are innate lymphocytes that recognize glycolipids expressed by the MHC Class I-like CD1d molecule [142]. NKTs actively traffic to neuroblastoma tissues and destroy CD1d-positive, cancer supporting tumor-associated macrophages (TAMs), and the presence of NKTs in neuroblastoma is associated with improved outcomes [142–146]. GD2-CARs can be expressed in NKTs. The generated CAR-NKTs can destroy both GD2-positive neuroblasts and CD1d-positive TAMs resulting in a potent antitumor effect in an aggressive metastatic neuroblastoma model in humanized mice [147].

Taken into account the above parameter, our group is currently developing and testing the 8B6 mAb based CARs expressed against neuroblastoma to find a potent and safe immunotherapeutic approach for patients. As trafficking may improve at the tumor sites,* O*-acetyl-GD2-CAR expressing effector cells may reach areas in the central nervous system (CNS) with* O*-acetyl-GD2-expressing resulting in on-target/off-tumor side effects. The two clinical studies previously testing GD2-CARs (NCT00085930; NCT01822652) have not shown thus far significant CNS-specific side effects and the final confirmation of safety for* O*-acetyl-GD2-CARs will also have to come from Phase 1 studies testing distinct effector lymphocyte subsets.

## 10. Conclusion

To date, passive immunotherapy with anti-GD2 therapeutic antibody in patients with neuroblastoma is the first successful glycan-targeted immunotherapy. Thus, targeting glycan antigens is a feasible therapeutic option for cancer immunotherapy, beside many unknown regarding their biological functions. The absence of* O*-acetyl-GD2 expression on nerve fibers and the lack of allodynic properties of anti-GD2 antibodies, which are believed to play a major role in mediating anti-GD2 therapeutic antibodies dose-limiting side effects, provide an important rationale for the clinical application of immunotherapeutic strategies in patients with* O*-acetyl-GD2-expressing tumors. Better tolerance shall allow the development of next generation of targeted immunotherapies. For example, the therapeutic antibodies can be engineered into more potent molecules. In this regard, constant efforts are needed to assess the functions of this particular antigen in tumor cells, since this information should provide a mechanistic basis for the optimization of the rational design of anti-*O*-acetyl-GD2 therapeutic antibodies. Other applications include CAR cell therapy. Here, several parameters remain to be defined given that there is a fine interplay between the scFv, spacer, transmembrane, signaling domains, and the effector cell type. The current strategies widely used, however, may not be sufficient to account for all of these variables in the quest of finding the best* O*-acetyl-GD2-CAR, and large-scale approaches are necessary to find the construct in a specific effector cell type with the most potent antitumor efficacy. Lastly, for vaccine strategies, it remains necessary to design immunization protocols that allow a high-affinity IgG response for* O*-acetyl-GD2 because of their weak immunogenicity. To this end, anti-idiotypic mAbs represent an attractive approach.

## Figures and Tables

**Table 1:** Relevant characteristic of *O*-acetyl-GD2 ganglioside as a cancer antigen according to Cheever et al. [26].

Criteria	Data on *O*-acetyl-GD2
Therapeutic function	Preclinical data showing that anti-*O*-acetyl-GD2 mAbs induce tumor cell death by immunological and nonimmunological mechanisms [39, 80, 87, 104].
Immunogenicity	Poorly immunogenic [95].
Oncogenicity	Increased expression in adult and pediatric solid tumors, to be determined with a clear association with oncogenic process [39, 40, 65, 87].
Expression level and positive cell	Overexpressed in cancer with little or no expression in normal tissues [39].
Stem cell expression	Expression on cancer with cancer stem cell issue such as glioblastoma, but without information about putative stem cells [87].
Number of patients with antigen-positive cancers	High level of expression in >70% of patients with a particular cancer type [39, 87].
Number of epitopes	Short antigenic segment with one or few epitopes [95].
Cellular location of expression	Expressed on the cell surface [39, 87] with little or no circulating antigen [104].
